# Research Progress on the Regulatory Mechanisms of Salt-Stress Response and Functional Genes in *Populus*

**DOI:** 10.3390/cimb48070684

**Published:** 2026-07-03

**Authors:** Peiyang He, Hanyang Cai

**Affiliations:** College of Life Sciences, Fujian Agriculture and Forestry University, Fuzhou 350002, China

**Keywords:** *Populus*, salt stress, molecular mechanism, ion homeostasis, transcription factor

## Abstract

Soil salinization represents one of the most severe abiotic constraints on global forest productivity. *Populus*, the most widely cultivated fast-growing timber tree and a premier model woody plant, exhibits striking intrageneric variation in salt tolerance—from the extremely halophytic *Populus euphratica* to highly salt-sensitive cultivated clones. Understanding the molecular basis of this variation has profound implications for saline–alkali land reclamation and salt-tolerant variety breeding. This review systematically synthesizes current knowledge on *Populus* salt-stress responses, covering three primary injury mechanisms (osmotic stress, ionic toxicity, and oxidative damage) and the corresponding physiological countermeasures. We further survey functional genes across four major categories: ion transporters, osmotic-adjustment enzymes, antioxidant-defense components, and transcription factors. Crucially, we extend beyond the herbaceous-plant paradigm by examining salt-tolerance strategies that are specific to the woody architecture of *Populus*: long-distance radial and axial Na^+^ transport through tall stems, salt sequestration in senescent bark and wood parenchyma, and deep-root ion exclusion strategies. Comparative insights from other woody genera are incorporated to highlight convergent and divergent mechanisms. On this basis, we propose an integrated multi-level regulatory model in which Na^+^ compartmentalization/efflux serves as the core, ROS homeostasis as the key regulatory axis, and osmotic adjustment as the auxiliary strategy. Outstanding challenges—including unresolved primary salt-signal perception, insufficient pathway integration, and limited in planta gene-function verification—are critically assessed, and future research priorities encompassing multi-omics integration, CRISPR-based gene editing, and natural-population genomics are outlined.

## 1. Introduction

Soil salinization is one of the foremost abiotic stress factors constraining the sustainable development of global agriculture and forestry. Approximately 20% of arable land and over 50% of irrigated farmland are threatened by salinity damage, and the affected area continues to expand owing to climate change and unsustainable irrigation practices [1]. In this context, the development of salt-tolerant plant varieties has become a research priority of considerable scientific and economic importance. Salt stress imposes osmotic stress, ionic toxicity, and secondary oxidative damage on plant cells, activating a complex multi-tiered signaling and response network spanning from ion transport and osmotic adjustment to transcriptional reprogramming [2,3].

Salt stress exerts a tripartite deleterious effect on plant physiology. The initial osmotic phase reduces soil water potential, rapidly impeding root water uptake and triggering stomatal closure, which in turn limits photosynthetic carbon assimilation and growth. The subsequent ionic phase is characterized by the progressive accumulation of Na^+^ and Cl^−^ in the cytosol to toxic concentrations, disrupting enzyme activity, protein synthesis, and membrane integrity, while simultaneously impairing the uptake of essential nutrients such as K^+^, Ca^2+^, and NO^3−^ through competitive ion transport. These ionic imbalances, combined with stomatal limitation of CO_2_ fixation, lead to the overproduction of reactive oxygen species (ROS) including superoxide (O^2−^), hydrogen peroxide (H_2_O_2_), and hydroxyl radicals (OH^−^), causing oxidative damage to lipids, proteins, and nucleic acids—constituting the third, oxidative phase of salt stress [2,4]. Plants have evolved a coordinated suite of adaptive strategies to counter these stresses, including osmotic adjustment through compatible solute accumulation, ion homeostasis via regulated transport and compartmentalization, and enzymatic and non-enzymatic antioxidant defense systems [5,6]. The molecular basis of these mechanisms has been extensively characterized in herbaceous model plants such as *Arabidopsis thaliana* and rice; however, woody perennials such as *Populus* present additional structural and physiological features—tall stature, perennial life history, extensive secondary growth, and deep root systems—that modulate salt-stress responses in ways not captured by herbaceous models.

*Populus*, belonging to the family Salicaceae, is the most widely distributed fast-growing timber genus and is established as the premier model system for molecular biology research in forestry. Among its members, *Populus euphratica* Olivier is a remarkable halophyte capable of surviving in soils with up to 5% NaCl, whereas most cultivated clones are highly salt-sensitive. This intrageneric contrast makes *Populus* an exceptionally tractable system for dissecting the genetic architecture of salt tolerance in woody plants. Earlier reviews have provided a broad overview of the physiological and molecular responses of *Populus* to salt stress [7], laying important groundwork that the present review extends by incorporating recent gene-function studies and an explicit woody-plant-specific perspective.

The completion of the *P. trichocarpa* reference genome [8] and subsequent multi-omics studies have propelled rapid advances in our understanding of *Populus* salt tolerance at the molecular level. However, most mechanistic discussions have been framed within a herbaceous-plant paradigm, overlooking physiological and structural features unique to woody species—such as tall stature with long-distance ion transport, radial growth generating new xylem and phloem annually, senescent bark accumulating salts, and deep-root systems that differentially encounter soil salt gradients. A comprehensive review that integrates the herbaceous-plant molecular framework with woody-plant-specific characteristics is therefore overdue.

This review systematically summarizes recent advances in the molecular mechanisms of *Populus* salt tolerance, with explicit attention to woody-plant-specific mechanisms and a comparative perspective spanning other arboreal taxa. We propose an integrated multi-level regulatory model and identify outstanding scientific challenges alongside promising research directions, aiming to provide a theoretical foundation and technical insights for molecular breeding of salt-tolerant. Specifically, this review aims to: (i) provide a comprehensive catalog of functionally validated salt-tolerance genes in *Populus*, organized by functional category—ion transporters, osmotic regulators, antioxidant enzymes, and transcription factors; (ii) critically evaluate the extent to which molecular mechanisms characterized in herbaceous model systems apply to, or require modification for, woody perennials; (iii) synthesize these findings into an integrated multi-level regulatory model that unifies ion homeostasis, osmotic adjustment, ROS detoxification, and hormone signaling under a hierarchical framework; and (iv) identify critical knowledge gaps and propose experimentally tractable research priorities that leverage emerging genomic and gene-editing tools. While a recent review has addressed salt, drought, and heavy-metal stress responses in *Populus alba* and its hybrids [9], the present review is distinguished by its specific focus on woody-plant-specific anatomical and physiological characteristics of *Populus*—including long-distance Na^+^ transport through tall stems, bark-mediated salt sequestration, and deep-root ion exclusion—which are absent from herbaceous model systems and not covered in depth by prior reviews. Furthermore, this review explicitly proposes an integrated multi-level regulatory model, a conceptual framework that hierarchically connects Na^+^ compartmentalization/efflux (core), ROS homeostasis (key regulatory axis), and osmotic adjustment (auxiliary strategy) with transcriptional co-regulation by multiple transcription factor families.

A substantial number of previously published reviews on *Populus* salt, drought, and heavy-metal stress responses were identified during the search process and critically evaluated. While these earlier syntheses provided valuable groundwork, the present review is distinguished by its exclusive focus on woody-plant-specific anatomical and physiological features—tall-stature ion transport, secondary growth dynamics, bark-mediated ion sequestration, and ectomycorrhizal symbiosis—that are systematically underrepresented in both herbaceous model-centric reviews and prior broad-stress surveys of *Populus*.

## 2. Physiological Responses of *Populus* to Salt Stress

Through long-term adaptive evolution, *Populus* has developed a multi-level physiological response system to salt stress, integrating ion homeostasis regulation, osmotic adjustment, antioxidant defense, and phytohormone signaling to collectively mitigate the triple damage of osmotic stress, ionic toxicity, and oxidative damage.

### 2.1. Ion Homeostasis Regulation

Maintaining intracellular ion homeostasis is central to *Populus* salt tolerance, achieved through coordinated regulation of Na^+^/K^+^ balance and suppression of cytoplasmic Na^+^ toxicity via multiple complementary pathways.

The Salt Overly Sensitive (SOS) signaling pathway constitutes the primary Na^+^-exclusion mechanism and is highly conserved in *P. euphratica*. Upon salt perception, a transient rise in cytoplasmic Ca^2+^ activates Calcineurin B-Like (CBL) proteins and their interacting kinases (CIPKs), forming a complex that phosphorylates and activates the plasma-membrane Na^+^/H^+^ antiporter SOS1, thereby driving extracellular Na^+^ efflux from root cells [10]. Vacuolar Na^+^ sequestration via NHX antiporters represents a complementary strategy: *PeNHX1*, *PeNHX2*, and *PeNHX5* are constitutively highly expressed in leaves and are further up-regulated under salt stress, facilitating Na^+^ compartmentalization into the vacuole [11]. Members of the High-affinity K^+^ Transporter (HKT) family limit long-distance Na^+^ transport to the shoot by modulating xylem loading, thereby protecting photosynthetic tissues from ionic toxicity [12].

The CBL-CIPK network further participates in ion homeostasis through regulation of multiple transport proteins. *PeCBL4* interacts with *PeCIPK24* and *PeCIPK26*, activating SOS1-mediated Na^+^ efflux under salt stress [13]. Calcium-dependent protein kinases *PeCPK7* and *PePKS5* enhance Na^+^ efflux and K^+^ influx in *Arabidopsis* root cells when overexpressed, maintaining K^+^/Na^+^ balance and improving salt tolerance [14].

### 2.2. Osmotic Adjustment

Osmotic adjustment is a critical strategy for counteracting salt-stress-induced water deficit. *Populus* accumulates compatible solutes—including proline, glycine betaine, soluble sugars (sucrose, trehalose), and raffinose-series oligosaccharides—to lower cellular osmotic potential, maintain turgor pressure, and stabilize membranes and enzymes.

Proline is the predominant osmoprotectant under salt stress; its content in *P. euphratica* leaves rises progressively with increasing NaCl concentration, closely correlated with induction of pyrroline-5-carboxylate synthetase (P5CS) gene expression [15]. Galactinol synthase genes *GolS1* and *GolS2* are markedly induced by salt treatment, enhancing synthesis of galactitol and raffinose and expanding the repertoire of osmoprotectants [16]. Betaine aldehyde dehydrogenase (PeBADH) and choline monooxygenase (PeCMO) act synergistically to sustain betaine biosynthesis, with PeCMO transcript levels increasing more than eight-fold in roots under salt stress [17]. Soluble-sugar synthesis genes *PeSPS* (sucrose phosphate synthase) and *PeTPS* (trehalose-6-phosphate synthase) are likewise significantly up-regulated, collectively enhancing cellular osmotic capacity [18]. Notably, the hybrid *P. talassica* × *P. euphratica* coordinates osmoprotectant synthesis across aboveground and belowground tissues at low salinity, but this coordinated response is significantly impaired at high salt concentrations, illustrating that osmoregulatory efficiency is tightly constrained by the severity of salt stress [19].

### 2.3. Antioxidant Defense

Salt stress triggers overproduction of reactive oxygen species (ROS), which cause oxidative damage to membranes, proteins, and nucleic acids. *Populus* counters this challenge through a multi-tiered antioxidant system comprising both enzymatic and non-enzymatic components.

The enzymatic antioxidant cascade is anchored by superoxide dismutase (SOD), which converts O^2−^ to H_2_O_2_; catalase (CAT) and peroxidase (POD) then reduce H_2_O_2_ to H_2_O; ascorbate peroxidase (APX) and glutathione peroxidase (GPX) further contribute to redox detoxification. In 84K *Populus*, antioxidant enzyme activities initially increase and then decline with escalating NaCl concentration, indicating that moderate salinity is manageable whereas severe salinity overwhelms enzymatic capacity [20]. Tissue-specific transcriptomics revealed that root redox-related genes are most significantly enriched, designating the root as the primary ROS-scavenging organ [21].

Glutathione S-transferase *PeGSTU58*, localized in both the cytoplasm and nucleus, is up-regulated under salt stress. Its overexpression in transgenic *Arabidopsis* elevates the activities of SOD, POD, CAT, and GST while up-regulating stress-responsive genes *DREB2A* and *COR47* [22]. Overexpression of the peroxisome-proliferation gene *PePEX11* reduces H_2_O_2_ accumulation, highlighting a peroxisome-mediated contribution to ROS metabolism [23]. Non-enzymatic antioxidants—glutathione (GSH), ascorbic acid (ASA), and flavonoids—directly scavenge ROS and interact synergistically with the enzymatic system to maintain redox homeostasis [20].

## 3. Woody-Plant-Specific Mechanisms of Salt Tolerance in *Populus*

The discussion of plant salt tolerance has historically been dominated by findings from the herbaceous model species *Arabidopsis thaliana* and cereal crops. While the core ion-homeostasis and osmotic-adjustment pathways are largely conserved, *Populus* and other woody plants possess structural and physiological characteristics that generate both unique challenges and additional adaptive solutions unavailable to short-lived annuals. These wood-specific mechanisms deserve explicit consideration in any comprehensive review of *Populus* salt tolerance.

### 3.1. Long-Distance Ion Transport in Tall Trees

In tall trees such as *Populus*, Na^+^ absorbed by root cells must traverse a substantially greater axial distance to reach photosynthetically active canopy tissues compared with herbaceous plants. This long-distance transport is mediated primarily through the xylem stream, making the regulation of xylem Na^+^ loading a critical checkpoint for salt tolerance. HKT1-type transporters, which retrieve Na^+^ from the xylem apoplast back into surrounding parenchyma cells, are therefore of particular physiological importance in *Populus*. Overexpression of *PeHKT1;1* significantly reduces Na^+^ accumulation in leaves of transgenic *Populus* [24], consistent with a “recirculation” model in which Na^+^ unloaded by xylem parenchyma HKTs is redirected to the phloem for basipetal retranslocation to roots.

Radial ion transport across multiple wood rings presents an additional complexity absent in herbaceous species. As secondary xylem (wood) is formed annually through cambial activity, Na^+^ stored in the symplast of living ray parenchyma and axial parenchyma of older wood can act as a long-term sink, buffering fluctuations in apoplastic salt concentration. This buffering capacity increases with tree maturation, potentially explaining why adult *P. euphratica* trees tolerate higher salt concentrations than seedlings. The molecular regulation of wood-parenchyma Na^+^/H^+^ antiporters (tonoplast NHX and vacuolar H^+^-ATPase/PPase) in this context remains largely unexplored and represents a priority for future research as it remains insufficiently characterized. While some progress has been made—DNA methylation changes under salt stress have been reported in *Populus* [25], non-coding RNAs (miRNAs, lncRNAs) have been identified as regulators of salt tolerance in multiple *Populus* species [26], and ubiquitylation and phosphorylation have been documented in *Populus* stress responses [27,28]—systematic and integrated multi-layered epigenomic profiling specifically under salt stress remains limited. Evidence from other plant systems further underscores the importance of these regulatory layers in salt-tolerance responses [29,30,31]. Comprehensive profiling in *Populus* is likely to reveal additional regulatory dimensions not captured by transcriptomics alone.

### 3.2. Salt Sequestration in Bark and Senescent Tissues

A distinctive feature of woody plants is the presence of bark—comprising the phloem, cortex, and periderm—as a substantial carbon and mineral reservoir. In salt-stressed *Populus*, the outer bark and older phloem tissues have been reported to accumulate Na^+^ and Cl^−^ at concentrations substantially higher than those in living mesophyll cells [21], suggesting that these metabolically less active tissues serve as a salt dump protecting the photosynthetic canopy. This mechanism is analogous to the salt-bladder strategy employed by halophytes such as *Atriplex* and *Mesembryanthemum*, but operates via a fundamentally different anatomical compartment—the secondary phloem and bark parenchyma. Periodic bark shedding in *Populus* may therefore constitute a passive salt-excretion mechanism, although direct experimental evidence for this hypothesis is lacking and warrants investigation through isotopic-tracer studies combined with laser-ablation ICP-MS.

Comparisons with other broad-leaved trees support the importance of bark salt storage: *Quercus suber* (cork oak) accumulates substantial Na^+^ in its thick bark under saline irrigation [32], and the mangrove *Avicennia marina* secretes excess salt via specialized bark-associated salt glands [33]. Although *Populus* lacks dedicated salt glands, the principle of peripheral-tissue salt storage appears to be a broadly convergent woody-plant strategy.

### 3.3. Deep Root Systems and Soil Salt Avoidance

The deep, extensive root architecture of *Populus* confers a spatial advantage over herbaceous crops: deep roots can access water from below the saline zone, effectively practicing salt avoidance by reducing total Na^+^ uptake. *P. euphratica* in its natural riparian habitat of the Tarim Basin exploits this strategy to access deep freshwater reserves during drought, while surface soils are highly saline [19]. Root plasticity—including preferential growth into low-salinity sub-horizons—is regulated by root gravitropism and hydrotropism pathways. *PagMYB73A* promotes adventitious root elongation in 84K *Populus* under salt stress [34], and the hormonal control of root architecture under salinity remains an active and promising research area.

### 3.4. Mycorrhizal Associations and Salt Tolerance

Mycorrhizal associations—ubiquitous in forest trees but rare or absent in most herbaceous model plants [35]—further augment salt tolerance of *Populus* by improving water and nutrient acquisition, modifying rhizosphere chemistry, and directly attenuating Na^+^ uptake [36]. Ectomycorrhizal fungi associated with *P. tremula* and *P. euphratica* have been shown to reduce foliar Na^+^ concentrations under salt stress [37,38], pointing to a tripartite root–mycorrhiza–salt-tolerance interaction that is an intrinsic component of woody-plant salt biology but has no parallel in *Arabidopsis* or rice research.

### 3.5. Comparative Perspectives with Other Woody Species

Comparing *Populus* with other gymnospermous and angiospermous trees illuminates both shared principles and genus-specific adaptations. In *Pinus* (conifers), salt tolerance is achieved primarily through tight root exclusion and accumulation of polyols (mannitol, pinitol) as osmoprotectants, with NHX vacuolar antiporters also playing important roles in the needles [39]. Unlike *Populus*, conifer needles possess limited vacuolar volume for Na^+^ storage, and thick cuticular waxes reduce salt spray absorption—a relevant adaptation in coastal *Pinus* species. In *Quercus* (oaks), transcriptomic responses to salinity share core components with *Populus* (SOS pathway induction, ERF and WRKY activation) but show markedly lower induction of NHX and HKT genes, consistent with the relatively lower salt tolerance of most oak species [36]. The extreme salt tolerance of *Tamarix* (tamarisk)—a dicotyledonous tree that can withstand NaCl concentrations exceeding 3%—is achieved through specialized salt glands on leaves and stems that actively excrete Na^+^ and Cl^−^; transcriptomic studies have identified WRKY and MYB transcription factors among the differentially expressed genes in *Tamarix* under salt stress, suggesting potential convergence with *Populus* stress-response pathways. These cross-species comparisons underscore the value of a comparative tree biology framework for identifying mechanistic innovations specific to *Populus* and for pinpointing candidate genes that could be transferred to improve salt tolerance in other timber species.

## 4. Functional Genes Related to Salt Tolerance in *Populus*

Salt-tolerance functional genes in *Populus* can be classified into four major categories based on their molecular functions: ion-transport genes, osmotic-adjustment genes, antioxidant-defense genes, and transcription-factor genes. These categories are discussed below and are catalogued in Table 1, Table 2, Table 3 and Table 4.

### 4.1. Ion Transport-Related Genes

Ion-transport genes orchestrate the efflux, vacuolar sequestration, and long-distance distribution of Na^+^, as well as the uptake and retention of K^+^, to maintain a high intracellular K^+^/Na^+^ ratio and mitigate Na^+^ toxicity (Table 1).

The NHX family serves as the principal executor of vacuolar Na^+^ sequestration. *PeNHX1* and *PeNHX5* are constitutively up-regulated in leaves under salt stress and are responsible for pumping cytoplasmic Na^+^ into vacuoles; *PeNHX2* exhibits differential tissue-specific expression, reflecting functional specialization across organs [11,21]. Core SOS-pathway genes—*PeSOS1*, *PeSOS2* (CIPK24), and *PeSOS3* (CBL4)—form a conserved signaling cascade that phosphorylates and activates the plasma-membrane Na^+^/H^+^ antiporter SOS1 to drive Na^+^ efflux [20,40]. HKT1-type transporters (*PeHKT1;1*, *PeHKT1;5*) limit xylem Na^+^ loading and thereby protect leaf photosynthetic machinery from ionic toxicity [8,24]—a function of heightened importance in the tall-stature context discussed in Section 3.1. H^+^-ATPase genes *PeHA4* and *PagHA2* are induced within 12 h of salt treatment, maintaining membrane potential polarization to suppress K^+^ efflux and energize Na^+^/H^+^ antiport [41]. Overexpression of phospholipase D *PePLD8* further improves salt tolerance by modulating K^+^/Na^+^ flux and ROS homeostasis, revealing a novel lipid-signaling interface [39].

**Table 1:** Ion transport-related genes associated with salt tolerance in *Populus*.

Gene Family/Type	Representative Genes	Subcellular Localization	Main Function and Mechanism	Refs
**Ion Transport**	NHX family	*PeNHX1*, *PeNHX2*, *PeNHX5*	Tonoplast (vacuolar membrane); Na^+^ compartmentalization into vacuoles; reduce cytoplasmic Na^+^ toxicity	[11,21]
	SOS pathway core genes	*PeSOS1*, *PeSOS2 (CIPK24)*, *PeSOS3 (CBL4)*	Plasma membrane (SOS1); cytoplasm (SOS2/SOS3); SOS1 mediates Na^+^ efflux; SOS2-SOS3 complex phosphorylates and activates SOS1	[20,42]
	HKT family	*PeHKT1;1*, *PeHKT1;5*	Xylem parenchyma plasma membrane; retrieve Na^+^ from xylem apoplast; limit long-distance Na^+^ transport to shoot	[8,24]
	H^+^-ATPase genes	*PeHA4*, *PagHA2*	Plasma membrane; provide proton motive force for Na^+^/H^+^ antiport; maintain membrane potential to inhibit K^+^ efflux	[43]
	Phospholipase D gene	*PePLD8*	Plasma membrane/cytoplasm; modulate K^+^/Na^+^ flux and ROS homeostasis via lipid signaling	[35]

### 4.2. Osmotic Adjustment-Related Genes

Osmotic-adjustment genes control the biosynthesis of compatible solutes and thereby determine the capacity to counter salt-induced water deficit (Table 2). *P5CS*, encoding pyrroline-5-carboxylate synthetase, is the rate-limiting enzyme of proline biosynthesis and is significantly up-regulated in salt-stressed *P. euphratica* [15]. Galactinol synthases *GolS1* and *GolS2* catalyze galactitol and raffinose synthesis [16]. *PeCMO* and *PeBADH* collaboratively catalyze betaine biosynthesis [17]. *PeSPS* and *PeTPS* promote accumulation of sucrose and trehalose, respectively [18].

**Table 2:** Osmotic adjustment-related genes associated with salt tolerance in *Populus*.

Gene Subcategory	Representative Genes	Main Function and Mechanism	Refs
Proline synthesis	*P5CS*	Rate-limiting enzyme of proline biosynthesis; up-regulated under salt stress; proline acts as an osmoprotectant and ROS scavenger	[15]
Galactinol/raffinose synthesis	*GolS1*, *GolS2*	Catalyze galactitol and raffinose synthesis; enhance osmotic capacity and freezing tolerance	[16]
Betaine synthesis	*PeCMO*, *PeBADH*	Sequential catalysis of choline → betaine aldehyde (CMO) and betaine aldehyde → betaine (BADH); PeCMO induced >8-fold in roots under salt	[17]
Soluble sugar synthesis	*PeSPS*, *PeTPS*	Catalyze sucrose and trehalose synthesis; promote soluble sugar accumulation; TPS products also act as signaling molecules	[18]

### 4.3. Antioxidant-Defense Genes

Antioxidant genes are activated in response to salt-induced ROS overproduction, collectively maintaining cellular redox homeostasis (Table 3). Core antioxidant-enzyme genes—*PeSOD*, *PePOD*, *PeCAT*, *PeAPX*, and *PeGPX*—show pronounced root-enriched up-regulation in tissue-specific transcriptomic data, consistent with the root acting as the primary ROS-scavenging organ [44]. *PeGSTU58* plays a multifaceted protective role, enhancing antioxidant enzyme activities and inducing stress-responsive genes [22]. *PePEX11* regulates peroxisome proliferation and reduces H_2_O_2_ under salt stress [23]. *PagGRXC9* strengthens glutathione-mediated redox buffering [40]. In contrast, *PeGRP2* acts as a negative regulator whose overexpression increases salt sensitivity by disrupting photosynthesis, Na^+^ homeostasis, and ROS balance [45].

**Table 3:** Antioxidant-defense genes associated with salt tolerance in *Populus*.

Gene Subcategory	Representative Genes	Main Function and Mechanism	Refs
Core antioxidant enzyme genes	*PeSOD*, *PePOD*, *PeCAT*, *PeAPX*, *PeGPX*	Encode SOD/POD/CAT/APX/GPX; constitute primary enzymatic ROS scavenging system; most significantly enriched in roots	[36]
Glutathione S-transferase	*PeGSTU58*	Enhances SOD/POD/CAT/GST activities in transgenic Arabidopsis; up-regulates DREB2A and COR47; multifunctional ROS homeostasis maintenance	[22]
Peroxisome-related gene	*PePEX11*	Regulates peroxisome proliferation and function; reduces H_2_O_2_ under salt stress; mediates peroxisomal ROS metabolism	[23]
Glutaredoxin gene	*PagGRXC9*	Regulates glutathione metabolic pathway; increases GSH content; enhances redox buffering capacity in 84K *Populus*	[19,37]
Negative regulator	*PeGRP2*	Overexpression disrupts photosynthesis, Na^+^ homeostasis, and ROS balance, increasing salt sensitivity	[38]

### 4.4. Transcription Factor Genes

Transcription factors orchestrate broad transcriptional reprogramming during salt stress by binding cis-elements in the promoters of downstream functional genes (Table 4). In the *Populus* genome, multiple transcription-factor families—ERF, MYB, WRKY, NAC, and bZIP—have undergone significant expansion [46], and many members exhibit differential expression under salt stress.

*PeERF1* is a core positive regulator whose overexpression in 84K *Populus* markedly enhances antioxidant capacity and osmoregulatory competence [47]. *PdbERF109* and *RAP2L14* from the hybrid *P. davidiana* × *P. bolleana* likewise act as positive regulators; their silencing impairs root development and plant growth under salt stress [48]. *PagMYB73A* promotes adventitious root elongation and regulates stomatal density [34], while *PSAR1* (an MYB repressor) functions as a negative regulator through interaction with the ABA pathway [49]. *PaWRKY33* enhances tobacco salt tolerance and directly modulates the expression of SOS1 and NHX by binding W-box elements in their promoters [50]—a mechanistic link between the transcription factor hub and the ion-homeostasis module. NAC family members *PeNAC021*, *PeNAC072*, and *PagNAC55* are strongly induced by salt and participate in multi-process regulation encompassing osmotic adjustment, ion transport, and ROS scavenging [41,51]. bZIP factors *PebZIP33* and *PagbZIP23* bind abscisic acid response elements (ABREs) in the *PeSOS1* promoter, linking ABA-mediated stress signaling to the SOS ion-efflux pathway [52,53].

**Table 4:** Transcription factor genes associated with salt tolerance in *Populus*.

TF Family	Representative Genes	Main Function and Mechanism	Refs
ERF family	*PeERF1*, *PdbERF109*, *RAP2L14*	Positive regulators; PeERF1 enhances antioxidant activity and osmotic capacity; PdbERF109 and RAP2L14 promote salt tolerance and root development	[40,54]
MYB family	*PagMYB73A*, *PSAR1*	Bidirectional regulation: PagMYB73A promotes adventitious root elongation and stomatal density (positive); PSAR1 interacts with ABA signaling (negative)	[34,41]
WRKY family	*PaWRKY33*, *PeWRKY22*	PaWRKY33 binds W-box elements in SOS1 and NHX promoters; directly links TF hub to ion-homeostasis module; enhances antioxidant enzyme activities	[55]
NAC family	*PeNAC021*, *PeNAC072*, *PagNAC55*	Induced by salt; regulate P5CS (osmotic adjustment), NHX1 (ion transport), and SOD (antioxidant) genes; versatile salt-tolerance regulators	[44,56]
bZIP family	*PebZIP33*, *PagbZIP23*	Bind ABREs; regulate ABA-mediated stress signaling; PebZIP33 activates PeSOS1 promoter, bridging hormonal and ion-homeostasis pathways	[45,46]

## 5. An Integrated Multi-Level Regulatory Model of Salt Tolerance in *Populus*

Comprehensive synthesis of existing research demonstrates that salt tolerance in *Populus* is not governed by any single pathway but arises from the coordinated operation of a hierarchical multi-level regulatory network. Ion homeostasis—centered on Na^+^ vacuolar sequestration (NHX) and plasma-membrane efflux (SOS1)—forms the primary defensive core, ensuring that cytoplasmic Na^+^ concentrations remain below toxic thresholds. Osmotic adjustment, mediated by the coordinated synthesis of proline, betaine, raffinose, and soluble sugars, maintains cellular water potential and provides auxiliary support for stress adaptation. The antioxidant defense network—comprising enzymatic (SOD/CAT/APX/POD/GPX/GSTU) and non-enzymatic (GSH/ASA/flavonoids) components—eliminates ROS overproduction, preventing irreversible oxidative damage to macromolecules. These three functional modules are integrated by Ca^2+^ and ABA signaling nodes and are transcriptionally co-regulated by overlapping sets of ERF, MYB, WRKY, NAC, and bZIP transcription factors.

Critically, the woody architecture of *Populus* imposes an additional spatial dimension on this model: long-distance Na^+^ transport through tall stems demands HKT-mediated xylem retrieval at multiple points along the vascular axis, bark parenchyma provides a peripheral Na^+^ sequestration compartment supplementing vacuolar storage in leaf mesophyll, and deep roots can exploit spatial salt gradients in the soil profile. These tree-specific features are not merely additive but mechanistically integrated with the core molecular network: bark NHX expression is co-regulated by the same CBL-CIPK calcium-signaling module that controls SOS1 in roots, and wood-ray parenchyma tonoplast H^+^-ATPase activity energizes vacuolar Na^+^ sequestration using the same biochemical machinery as leaf mesophyll vacuoles.

The proposed multi-level model has important implications for the molecular breeding of salt-tolerant *Populus* varieties. First, the hierarchical organization of the model suggests that simultaneous modulation of the core Na^+^ exclusion module (SOS1/NHX/HKT) and the ROS regulatory axis (antioxidant enzymes/transcription factors) is likely to produce synergistic, rather than merely additive, improvements in salt tolerance—a prediction testable through combinatorial transgenic approaches. Second, recent genome-wide studies in *P. euphratica* have identified expanded gene families—including C2H2 zinc-finger proteins, aspartic proteases, and glycine-rich RNA-binding proteins—whose members exhibit salt-responsive expression patterns [57], providing a rich source of candidate genes for functional validation and breeding. Third, the cross-talk between hormone signaling pathways and the core salt-tolerance modules—particularly the ABA-dependent activation of MYB and NAC transcription factors and the JA-mediated modulation of antioxidant gene expression [42]—offers opportunities for hormone-based priming strategies to enhance salt tolerance in *Populus* plantations. Comparative analysis with salt-tolerant woody species outside Salicaceae—such as *Avicennia marina* (mangrove) and *Casuarina* spp.—further suggests that salt secretion via specialized glands and symbiotic nitrogen fixation under saline conditions represent alternative evolutionary strategies whose molecular underpinnings may inform orthogonal breeding approaches for *Populus*.

## 6. Current Challenges and Future Directions

Despite remarkable progress, several fundamental challenges persist in the study of *Populus* salt tolerance.

The primary molecular sensors of salt stress at the plasma membrane remain poorly characterized. The identity of the initial Na^+^ sensor and the mechanisms coupling ionic and osmotic perception to intracellular signaling cascades remain unclear, limiting our understanding of how salt signals are first decoded in *Populus* cells. Candidates emerging from *Arabidopsis* research—including the MSCL-like mechanosensitive channels and the OSCA family of hyperosmolality-gated Ca^2+^-permeable channels—require functional validation in *Populus*.

The temporal and environmental regulation of cross-talk among ion-homeostasis, osmotic-adjustment, and antioxidant pathways remains insufficiently characterized. In particular, the hormonal interaction network—ABA, ethylene, JA, and brassinosteroids—and its context-dependent modulation of salt-tolerance gene expression requires systematic investigation using high-resolution time-course transcriptomics and hormone-signaling mutants in *Populus*. The transcriptional regulation of salt-stress responses by these hormones has been documented in herbaceous species [43], but systematic characterization in *Populus* remains a priority.

Functional validation of salt-tolerance genes remains predominantly dependent on the *Arabidopsis* heterologous system, which lacks the woody architecture and perennial physiology of *Populus*. Direct in planta validation using stable transgenic *Populus* lines, combined with CRISPR/Cas9-mediated knockouts in *P. euphratica* itself, is essential for translating molecular discoveries into actionable breeding targets.

The roles of epigenetic regulation (DNA methylation, histone modifications), small and long non-coding RNAs (miRNAs, lncRNAs, circRNAs), and protein post-translational modifications (ubiquitylation, SUMOylation, phosphorylation) in the systemic salt-tolerance response of *Populus* have been largely unexplored. Multi-layered epigenomic profiling under salt stress is likely to reveal additional regulatory dimensions not captured by transcriptomics alone.

Looking forward, four research directions are prioritized: (i) multi-omics integration—combining transcriptomics, metabolomics, proteomics, and epigenomics within a systems-biology framework to reconstruct the full topology of the *Populus* CRISPR-based gene editing applications (including Cas9-mediated knockout, base editing, and prime editing) salt-response regulatory network; (ii) in *Populus* to conduct precise functional validation and rational engineering of salt-tolerance genes; (iii) population genomics of *P. euphratica* and its wild relatives to discover superior salt-tolerance alleles via genome-wide association studies; and (iv) translational breeding—leveraging marker-assisted selection, genetic transformation, and gene editing to develop elite *Populus* varieties adapted to saline–alkali soils, thereby supporting ecological restoration and sustainable forestry on salt-affected land.

## 7. Conclusions

This review provides a comprehensive synthesis of the molecular mechanisms underlying salt tolerance in *Populus*, organized within a hierarchical multi-level regulatory framework. The central thesis emerging from this synthesis is that salt tolerance in woody perennials cannot be reduced to any single molecular pathway—rather, it arises from the coordinated integration of ion homeostasis, osmotic adjustment, ROS detoxification, and hormone-mediated transcriptional regulation, all operating within a woody anatomical context that uniquely modulates these processes. The proposed model identifies Na^+^ vacuolar sequestration and plasma-membrane efflux as the primary defensive core, antioxidant networks as the central regulatory axis, and osmotic adjustment as a critical auxiliary support system, with CBL-CIPK calcium signaling and ABA-dependent transcription factors providing integrative control across all levels.

Several critical knowledge gaps remain that should guide future research. The identity of the primary Na^+^ sensor(s) in *Populus* has yet to be conclusively determined, and the structural basis of ion channel gating under salt stress awaits high-resolution characterization. The regulatory contributions of epigenetic modifications—DNA methylation, histone modification, and non-coding RNAs—to salt-responsive gene expression in *Populus* remain insufficiently characterized. The mechanistic basis of ectomycorrhiza-mediated salt tolerance improvement, though demonstrated physiologically, lacks molecular dissection. Bridging these gaps through the application of CRISPR-based gene editing, single-cell transcriptomics, and multi-omics integration holds transformative potential for both fundamental understanding and applied breeding of salt-tolerant forest trees.

In the broader context of global environmental change, the molecular breeding of salt-tolerant *Populus* varieties represents a strategic priority. Saline–alkali land occupies over 900 million hectares worldwide and continues to expand. As fast-growing woody perennials with well-developed genomic resources and established transformation protocols, salt-tolerant *Populus* cultivars offer a tangible pathway toward ecological restoration, carbon sequestration, and sustainable biomass production on marginal lands that are unsuitable for conventional agriculture.

## Data Availability

No new data were created or analyzed in this study. Data sharing is not applicable to this article.
